# Explainable Ensemble Machine Learning for the Prediction and Optimization of Pozzolanic Concrete Compressive Strength

**DOI:** 10.3390/polym18080933

**Published:** 2026-04-10

**Authors:** Sebghatullah Jueyendah, Elif Ağcakoca

**Affiliations:** Department of Civil Engineering, Sakarya University, Esentepe Campus, Serdivan 54050, Sakarya, Türkiye; sebghatullahjueyendah@gmail.com

**Keywords:** pozzolanic concrete, ensemble modeling, SHAP interpretability, multi-objective optimization, carbon footprint, compressive strength prediction

## Abstract

Pozzolanic concrete demonstrates intricate, highly nonlinear material interactions that pose significant challenges for the accurate prediction of compressive strength (CS). This study introduces a novel, interpretable ensemble machine learning (ML) framework for predicting CS based on 759 mixture records encompassing cement, aggregates, supplementary cementitious materials (pozzolans), water/binder (W/B), superplasticizer, water, and curing age. Descriptive analysis and ANOVA were used to identify key predictors, followed by an 80/20 train–test split with 10-fold cross-validation to ensure robust and generalizable modeling. To further enhance model reliability, 5% of outliers were removed using an isolation forest algorithm, after which data were normalized and ensemble hyperparameters optimized. Among the evaluated models, the extra trees algorithm with standard scaling demonstrated the most stable generalization, achieving a coefficient of determination (R^2^) of 0.978 and a root mean square error (RMSE) of 4.197 MPa on the test set, and R^2^ = 0.966 (RMSE = 5.053 MPa) under 10-fold cross-validation. Feature importance, SHAP, and partial dependence analyses consistently demonstrated that W/B, curing age, and cement are the principal determinants of CS. Finally, multi-objective optimization generated high-strength, low-impact mixtures, confirming the framework’s effectiveness as a transparent decision-support tool for performance- and sustainability-oriented pozzolanic concrete design. This study is novel in combining interpretable ensemble ML with multi-objective optimization to simultaneously achieve precise CS prediction and the formulation of sustainable, performance-optimized pozzolanic concrete mixtures.

## 1. Introduction

Concrete is the most widely used building material in the world, valued for its versatility, affordability, durability, and suitability for a variety of structural applications [1]. Global concrete production is projected to exceed 18 billion tons by 2050, reflecting growing global demand and its essential role in the development of contemporary infrastructure [2]. Concrete is a heterogeneous mixture consisting of water, cement, aggregates, and complementary chemical or mineral additives [3]. Cement hydration forms C-S-H as the primary strength-giving phase, while coarse and fine aggregates enhance compaction and provide a stable structural framework for overall strength and stability [4]. Concrete performance is governed by its mix proportions; although it exhibits high CS, its low tensile capacity necessitates reinforcement [5]. Concrete durability is primarily governed by environmental exposure conditions and the adequacy of curing, as proper curing ensures sufficient hydration and enhances long-term performance [6]. Hydration and strength in concrete are primarily influenced by the W/B, whereas superplasticizers adjust flow behavior, supplementary cementitious materials (SCMs) improve microstructural quality, and curing regimes shape the final mechanical performance [7]. The performance of concrete depends on its microstructural development during curing, while the fact that Portland cement production contributes nearly 8% of global CO_2_ emissions underscores the need for sustainable, low-carbon binder alternatives [8]. Pozzolanic concrete, formulated with SCMs and natural or industrial pozzolans, improves long-term durability while reducing cement consumption [9]. Pozzolanic reactions produce secondary C-S-H, which reduces permeability, increases durability, increases long-term strength, and reduces heat generation in mass concretes [10]. Integrating industrial by-products enhances the sustainability of concrete; nevertheless, predicting the CS of pozzolanic concrete remains challenging because of complex nonlinear interactions between components and the variable reactivity of pozzolanic materials, which conventional empirical models fail to fully capture [11]. To reduce the environmental burden associated with ordinary Portland cement (OPC), a range of low-carbon alternative binder systems has been developed [12]. Ground granulated blast furnace slag (GGBFS), an industrial by-product of iron and steel production, is widely used as SCMs to partially replace clinker and improve durability and long-term strength [13]. Fly ash (FA), a by-product of coal-fired power generation, possesses pozzolanic activity and enhances workability and later-age strength development [14]. Calcined clay, particularly in limestone–calcined clay cement (LC^3^) systems, facilitates substantial clinker reduction while preserving mechanical integrity, whereas alkali-activated materials (AAMs) derived from industrial by-products such as slag or fly ash provide high strength and chemical resistance with markedly lower carbon emissions than conventional OPC-based systems [15]. Relevant recent studies have been integrated to substantiate the efficacy of these sustainable binder alternatives.

This study employs ensemble ML models to predict the CS of pozzolanic concrete by effectively capturing its intricate nonlinear and multi-parameter relationships. ML enables the construction of computational frameworks that learn complex patterns directly from data, eliminating the reliance on explicitly defined rule-based models [16]. In material, civil, and structural engineering, ML is widely used to simulate material properties and complex system behavior, predict structural responses, and optimize design and maintenance strategies, thereby improving sustainability, efficiency, and productivity [17,18]. Ensemble learning methods, including extra trees, random forest, bagging, gradient boosting, adaboost, xgboost, and lightgbm, have demonstrated strong predictive capability in capturing the complex behavior of cementitious composites [19,20]. In 1959, Arthur Samuel described ML as a computational approach in which systems enhance their performance through experience rather than relying on explicitly programmed instructions [21]. The growing importance of artificial intelligence (AI) stems from its ability to generate predictions from data and model complex systems, while ML offers a data-driven approach to decision-making and pattern recognition [22]. As a branch of AI, ML learns patterns from data to improve prediction, comprising supervised, unsupervised, and reinforcement approaches, with supervised learning modeling labeled input–output relationships [23]. This study develops a comprehensive framework for the prediction and optimization of pozzolanic concrete by incorporating systematic outlier analysis, data normalization, and the assessment of seven ensemble learning models. Model interpretability is ensured using feature importance, SHAP analysis, partial dependency plots, and Taylor plots, while multi-objective optimization is applied to identify low-carbon, high-strength mixtures; among the models, ensemble ML shows the highest accuracy and robustness for sustainable concrete design. Ensemble ML models provide more reliable predictions of concrete strength than traditional approaches; therefore, in this study, we integrate ensemble-based prediction, interpretability analysis, and multi-objective optimization to promote sustainable pozzolanic concrete design [24]. AI enables data-driven prediction and optimization in civil engineering, with performance evaluated using standard metrics and interpretability assessed via SHAP and feature importance analysis. Olaiya et al. [24] systematically evaluated methods for enhancing the pozzolanic reactivity of industrial and agricultural by-products in concrete. Ahmed et al. [25] examined the fundamental chemical reactions that control the performance of pozzolanic concrete. Setina et al. [26] analyzed the effects of pozzolanic additives on the microstructure and chemical durability of concrete. Uzal et al. [27] evaluated the structural viability and mechanical performance of high-volume natural pozzolan concrete. Dembovska et al. [28] investigated how pozzolanic admixtures affect the development of the mechanical strength of high-performance concrete. McCarthy and Dyer [29] analyzed the chemical composition and structural features of pozzolans and associated pozzolanic materials in detail. Nwaokete et al. [30] assessed the environmental impact and sustainability performance of polypropylene fiber-reinforced pozzolanic concrete based on an extensive database analysis. Boomibalan et al. [31] explored the mechanical response and microstructural features of hybrid steel fiber–reinforced concrete incorporating pozzolanic and non-pozzolanic constituents. Abdelsattar et al. [9] investigated the use of ML combined with sensitivity analysis to enhance the mechanical performance and durability of pozzolanic concrete mixtures. Hassan et al. [32] predicted and modeled the CS of pozzolanic concrete with high accuracy using artificial neural network (ANN) models. Raju et al. [33] compared several ML approaches to predict the CS of pozzolanic concrete. Kao et al. [34] proposed a computer-based approach that combines ANNs with mixture classification to improve and optimize the mix design of pozzolanic concrete for engineering use. Khatoon et al. [35] integrated experimental results with hybrid ensemble ML methods to estimate the mechanical strength of fiber-reinforced geopolymer concrete. Sapkota et al. [36] used explainable LS-XGB along with optimization to predict CS of high-strength concrete. Nguyen and Phan [37] used random forest and adaptive gradient boosting to predict the CS of ultra-high-performance concrete (UHPC). Jueyendah et al. [38] predicted and modeled the compressive and flexural strengths of cement mortar using a support vector machine. Jueyendah and Humberto Martins [39] developed a combined SVR-RBF approach to predict and optimize the design and performance evaluation of welded beams. Mashhadban et al. [40] applied particle swarm optimization combined with ANN to predict and model the mechanical properties of FRSCC. Jueyendah et al. [41] demonstrated that nonlinear ML models exhibit markedly superior predictive accuracy relative to linear approaches in estimating cement mortar strength. Saha et al. [42] predicted the strength of SCC using ANN and multivariable regression analysis, demonstrating the applicability of data-driven modeling for performance evaluation. Ağcakoca et al. [43] developed a hybrid ML–FE framework using the CDP model to improve the prediction accuracy of cementitious composite behavior. Gencel et al. [44] created a fuzzy logic-based model to predict the properties of FRSCC. Nasir Amin et al. [45] examined the effect of waste glass powder on mortar flexural strength using ML, offering data-driven insights into its performance. Saridmir [46] predicted and modeled the CS of metakaolin-modified mortar using fuzzy logic and ANN and demonstrated their effectiveness in depicting the nonlinear behavior of materials. Siddique et al. [47] used an ANN to model and predict the CS of self-compacting concrete (SCC) and demonstrated its effectiveness in depicting the complex behavior of materials. Alahmari et al. [48] used a hybrid ML framework to model the CS of fiber-reinforced self-consolidating concrete. Kashem et al. [49] used data-driven hybrid methods, integrating SHAP and PDP analyses, to accurately predict the CS of UHPC. Jain et al. [50] utilized ML models to evaluate and predict the properties of construction materials. Guan et al. [51] employed ML techniques to evaluate the effect of glass powder on the CS of SCC. Jueyendah et al. [17] developed an interpretable hybrid ML–deep learning framework for predicting the CS of recycled powder mortar, enhancing predictive accuracy and model transparency.

### Research Novelty and Scientific Significance

Although ML shows promise in pozzolanic concrete, its application remains limited, with many studies neglecting essential steps such as advanced normalization, thorough outlier detection, and comprehensive interpretability analyses necessary for robust and generalizable models [9]. Interpretability frameworks such as feature importance, SHAP, and PDP analyses provide rigorous analytical transparency to ML predictions, while validation procedures, including cross-validation and residual diagnostics, ensure the methodological robustness and generalizability of the resulting models. Current materials research is increasingly focused on optimizing concrete mixtures to improve mechanical performance while minimizing environmental impacts. Metaheuristic algorithms such as differential evolution (DE), genetic algorithms (GA), moth-flame optimization (MFO), particle swarm optimization (PSO), and whale optimization (WOA) offer powerful tools for materials optimization, yet their integration with high-fidelity ML predictive models remains largely unexplored. To address these gaps, this study proposes a comprehensive ensemble ML framework, incorporating adaboost (AB), xgboost (XG), gradient boosting (GB), random forest (RF), bagging decision trees (BD), extra trees (ET), and lightgbm (LG), to predict the CS of pozzolanic concrete based on nine critical mixture parameters: cement, SCMs, pozzolans, fine and coarse aggregates, water/binder (W/B), superplasticizer, water, and curing age. The methodology combines ANOVA and statistical analysis-based significance analysis with multi-method outlier detection (modified Z-score, IQR, LOF, Z-score, DBSCAN, KNN distance, isolation forest, and one-class SVM) and a systematic evaluation of normalization techniques, including robust, standard, min–max, and max–abs scaling. The predictive performance of the ensemble models was evaluated using a suite of rigorous metrics, including ad percent bias (Pbias), adjusted R^2^ (AdjR^2^), centered root mean squared error (CRMSE), mean squared error (MSE), root mean squared error (RMSE), mean absolute error (MAE), median absolute error (MedAE), coefficient of determination (R^2^), standard deviation (SD), and Pearson correlation coefficient (PCC), with the ET model exhibiting superior robustness, accuracy, interpretability, and generalizability. Integrating ensemble ML models, normalization, and interpretability tools, this study enables accurate, transparent prediction of pozzolanic concrete strength, while metaheuristic optimization concurrently guides the design of high-performance, low-carbon mixtures.

## 2. Materials and Methods

**Figure 1:**
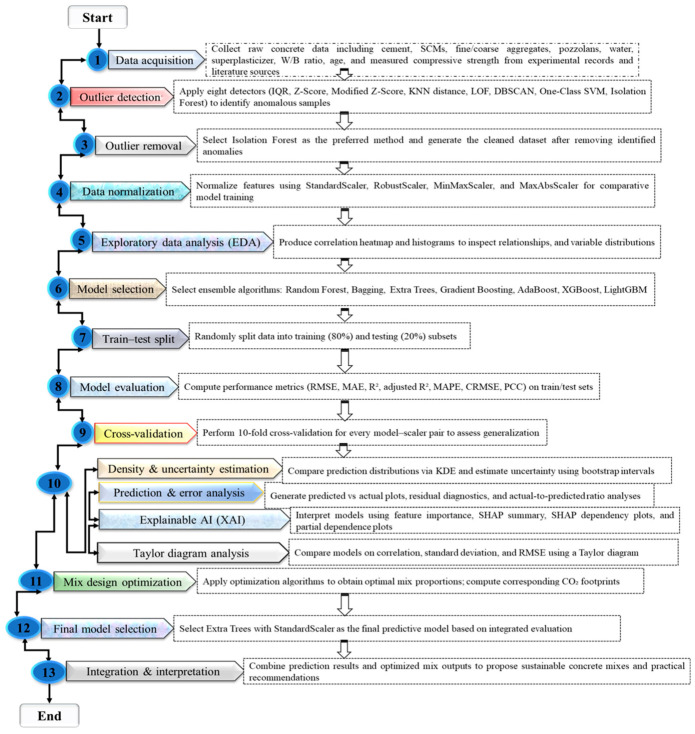
Research methodology framework.

A database of 759 pozzolanic concrete mixtures [52,53,54,55,56,57,58,59,60], with key mixture parameters as inputs and CS as output, was compiled to support predictive modeling and optimization. Descriptive statistics, ANOVA, and correlation analysis identified key predictors, and the dataset was split 80/20 with 10-fold cross-validation to ensure robust and generalizable model performance. Outliers were detected using modified Z-score, Z-score, IQR, DBSCAN, LOF, KNN distance, isolation forest, and one-class SVM, with isolation forest selected as the best method, eliminating 5% of the dataset. The dataset was then normalized using robust, min–max, standard, and max–abs scalers to improve model performance. ET, GB, AB, XG, RF, BD, and LG models underwent systematic hyperparameter tuning and were rigorously evaluated using a comprehensive suite of performance metrics, including R^2^, AdjR^2^, MedAE, Pbias, MSE, RMSE, MAE, CRMSE, SD, and PCC. Model interpretability was evaluated using feature importance, SHAP, and partial dependence analyses, while multi-objective optimization with GA, DE, PSO, MFO, and WOA determined mixture designs that maximize CS and minimize CO_2_ emissions. The research methodology implemented in this study is schematically illustrated in Figure 1.

**Figure 2:**
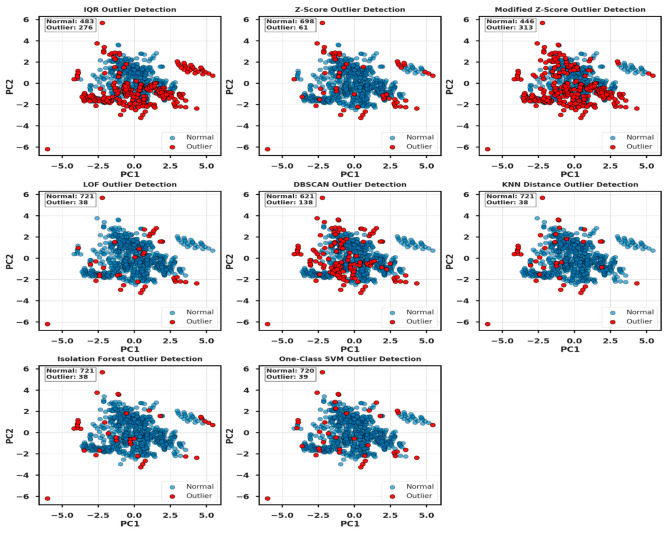
Comparison of outlier detection in the concrete dataset using eight different methods.

Figure 1 presents the workflow for modeling and optimizing pozzolanic concrete CS, with outlier detection, normalization, and exploratory analysis, and ET, with standard scaling selected for its accuracy and interpretability. Table 1 and Figure 2 provide a comparative analysis of eight statistical and ML techniques: IQR, Z-score, modified Z-score, LOF, DBSCAN, KNN distance, isolation forest, and one-class SVM, applied for outlier detection in the concrete dataset. Data analysis and model development were conducted in Python 3.11 using an integrated development environment, enabling efficient preprocessing, training, and evaluation. Python, supported by scientific libraries such as NumPy and Pandas, provided a robust and efficient computational environment for data processing, model development, evaluation, and visualization in this study [61,62].

**Figure 3:**
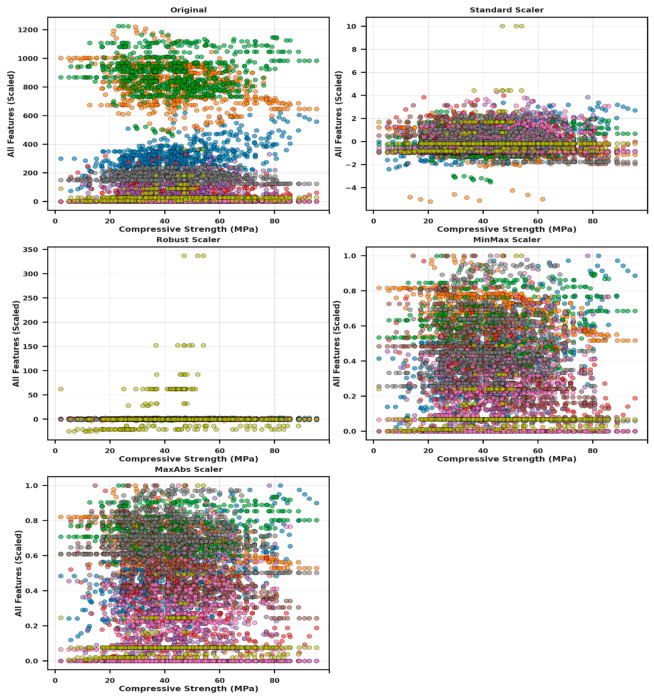
Distribution of input features relative to CS under five data-scaling strategies.

**Figure 4:**
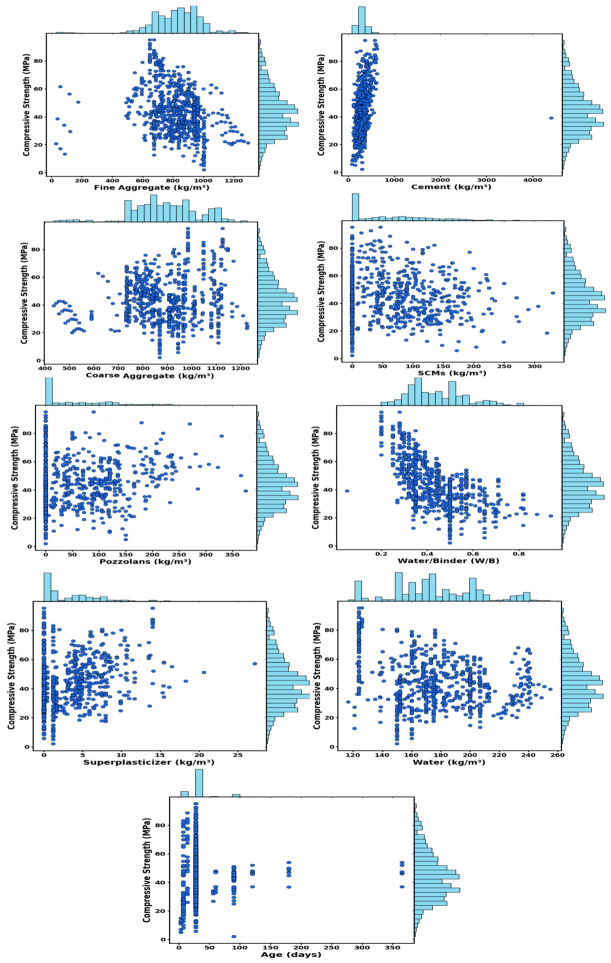
Bivariate relationships of CS with inputs.

Table 1 compares outlier detection methods on a 759-sample concrete dataset, showing that IQR and modified Z-score exclude 36–41% of data, DBSCAN flags ~18%, and LOF, KNN, isolation forest, and one-class SVM remove ~5%, with ensemble-based approaches effectively identifying both global and local outliers while minimizing data loss and preserving analysis integrity. Figure 2 presents eight outlier detection methods applied to real datasets on PC1 and PC2, showing that IQR, modified Z-score, and DBSCAN detect many anomalies, while LOF, KNN, one-class SVM, and isolation forest remove few samples, with isolation forest selected for balancing detection and data preservation; feature normalization was applied using min–max, robust, standard, and max–abs scalers to mitigate outlier effects. Figure 3 depicts the distributional behavior of all input features in relation to CS under five distinct data-scaling strategies, including the original unscaled dataset, standard scaler, robust scaler, min–max scaler, and max–abs scaler. The unscaled data display substantial variability and heterogeneous numerical ranges across features, whereas the scaled versions attenuate these disparities to varying degrees. Robust and standard scaler centralize the data, while max–abs and min–max scaler compress features into fixed ranges to improve consistency. The figure illustrates how each scaling method reshapes the feature space, thereby influencing the structure and comparability of the dataset for subsequent modeling. CS prediction incorporated key inputs, including aggregates, cement, SCMs, pozzolans, water, superplasticizer, W/B, and age, whose contributions were examined via ANOVA. As reported in Table 2, F-statistics and *p*-values (*p* < 0.05) denote the variables exerting statistically significant effects on CS [63]. ANOVA and descriptive statistics confirm that all variables significantly influence CS, with W/B and age being the most impactful, as summarized in Table 2. The dataset shows substantial variability in cement (mean = 301.01 kg/m^3^, SD = 95.03 kg/m^3^), which exhibits a significant effect on CS (F = 4.02, *p* < 0.001). Fine and coarse aggregates likewise display wide ranges and significantly influence CS (F = 4.67 and 6.78, *p* < 0.001). Pozzolans and SCMs significantly affected CS, with SCMs showing a statistically stronger effect (F = 2.10, *p* < 0.001) than pozzolans (F = 1.28, *p* = 0.016). The W/B exhibits the most pronounced statistical influence on CS (F = 16.88, *p* < 0.001), while water, superplasticizer, and age likewise present significant contributions to CS variability (*p* < 0.001). Figure 4 presents scatterplots with marginal histograms for each feature relative to CS.

Figure 4 presents scatterplots with marginal histograms showing that CS correlates strongly positively with cement and negatively with low W/B, while SCMs, aggregates, and pozzolans display weaker, more diffuse trends. This figure illustrates the heterogeneous effects of mixture components on compressive strength, with moderate superplasticizer influence and strength increasing, then stabilizing, over a wide age range.

**Figure 5:**
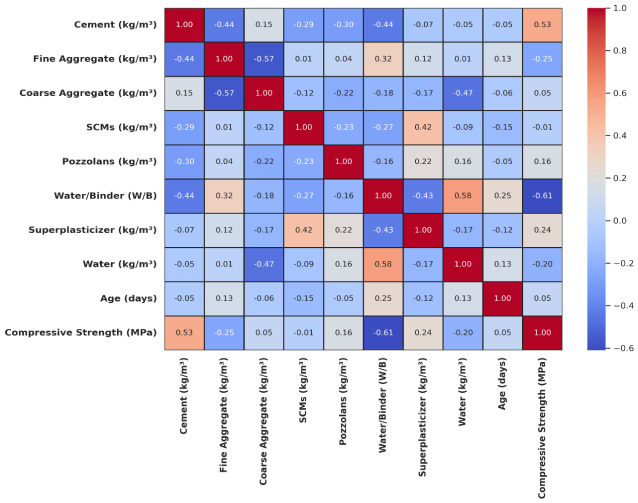
Correlation heatmap of input variables and CS.

All preprocessing procedures, including outlier detection and feature scaling, were applied exclusively to the training subset. The fitted transformations were then applied to the test set to prevent data leakage, ensuring unbiased evaluation of model performance and reliable assessment of generalization capability. Figure 5 shows the correlation heatmap of input variables and CS, highlighting key predictors and informing feature selection for improved modeling. Pozzolans (r = 0.16) and superplasticizer (r = 0.24) exhibit moderate positive correlations, while cement (r = 0.53) shows a strong positive correlation with CS. The fine aggregate (r = −0.25), W/B (r = −0.61), and water (r = −0.20) show negative associations with CS, with W/B exerting the strongest inverse effect, while cement remains the most influential positive contributor. The heat map identifies key variables affecting ML predictions, reveals potential multicollinearity between inputs, and validates the established material science relationships, thereby aiding feature selection and improving predictive modeling.

### 2.1. Ensemble Machine Learning Models

Ensemble ML models integrate multiple base learners to achieve higher prediction accuracy and robustness than individual models [64]. Ensemble methods combine multiple models to reduce overfitting, enhance generalizability, and capture complex nonlinear relationships, making them well-suited for civil and structural engineering applications [65]. Ensemble ML integrates multiple learners to enhance accuracy, robustness, and generalizability, with ET leveraging random splits to capture nonlinear relationships efficiently while reducing variance [66]. BD and RF use a set of bootstrapped decision trees with random feature selection, which reduces variance, improves generalizability, and increases robustness in modeling nonlinear data [67,68]. GB and AB form successive groups of weak learners that reweight misclassified examples and minimize residual errors to capture nonlinear interactions and improve prediction accuracy [69,70]. XG uses regular gradient boosting for fast and robust learning on large datasets with missing values, while LG uses leaf-centered tree growth to provide highly efficient and accurate large-scale modeling [71,72]. Ensemble models improve accuracy, robustness, and nonlinear modeling, supporting applications such as material property prediction, structural evaluation, maintenance planning, and risk assessment [73].

### 2.2. SHAP Analysis and Feature Importance

SHAP (SHapley Additive exPlanations) quantifies each feature’s marginal contribution to an ML prediction, with the SHAP value sign indicating its positive or negative effect on the outcome [74]. SHAP offers consistent, model-independent interpretation, capturing feature interactions and nonlinear effects, and highlights key factors influencing concrete strength and structural performance for data-driven design. Traditional feature importance provides overall impact measures that can be biased for correlated or nonlinear data, while SHAP increases transparency, interpretability, and practical relevance in engineering [75].

### 2.3. Partial Dependence Analysis

Partial dependency analysis (PDA) assesses the marginal effect of input features on predictions, reveals nonlinear relationships, and improves the interpretability of complex ensemble models [76]. PDA increases the interpretability and reliability of data-driven decisions and facilitates feature selection by systematically identifying influential variables. In civil and structural engineering, PDA connects predictive accuracy with interpretability to evaluate material proportions, loads, and environmental effects, supporting sensitivity analysis, optimization, and risk assessment [77].

### 2.4. Regression Model Performance Metrics

The performance of the regression model is evaluated using multiple statistical criteria to ensure its accuracy, reliability, and robustness [78]. R^2^ indicates the variance explained, adjusted R^2^ accounts for predictor number, RMSE and MSE emphasize large errors, MAE measures mean absolute error, and Pbias evaluates systematic prediction bias [79]. CRMSE quantifies random error after bias removal, SD measures observed variability, PCC evaluates linear correlation strength, and MedAE provides a robust median-based error less sensitive to outliers. These metrics provide a framework for evaluating regression models, summarized with definitions and formulations in Table 3.

### 2.5. Robust Statistical and Algorithmic Approaches for Outlier Identification

Outlier detection techniques in material, civil, and structural engineering are crucial for reducing noise caused by instrumentation, environmental variations, and human error in experimental and monitoring datasets [81]. The IQR method detects extreme values, while the modified Z-score and Z-score identify anomalies from the central tendency, aiding quality control in concrete, steel, and heterogeneous mixtures [82]. DBSCAN, LOF, and KNN distance detect anomalies, supporting damage detection, SHM noise filtering, and response isolation [83]. One-class SVM and isolation forest model normal structural behavior, detecting rare events such as early cracking or long-term bridge anomalies in large datasets [84]. These techniques enhance data integrity and predictive reliability in civil engineering by eliminating nonphysical records and sensor artifacts.

### 2.6. Feature Scaling

Feature scaling normalizes variable ranges to prevent bias, improve convergence, and increase prediction accuracy in civil and structural engineering applications [85]. The standard scaler centers the features and scales them by unit variance, increasing the comparability and stability of the model in predicting concrete strength and SHM [86]. The min–max scaler transforms data into a defined range and preserves the relative proportions of features for applications such as soil classification and NNs modeling, while the robust scaler uses the median and IQR to reduce the impact of outliers in heterogeneous materials and geotechnical measurements [87]. The max–abs scaler scales data by the maximum absolute value, preserving sign and dispersion, and, overall, increasing numerical stability and prediction reliability in civil engineering.

### 2.7. Metaheuristic Optimization

Optimization finds constrained solutions to improve performance, safety, and cost, addressing complex civil engineering challenges via meta-heuristic methods [88]. Differential evolution (DE) uses population differences to solve continuous problems and facilitates structural measurement, while particle swarm optimization (PSO), inspired by collective behavior, effectively addresses continuous and multi-objective tasks such as reinforcement layout and bridge design [89]. Genetic algorithms (GA) optimize complex domains in structural design, concrete mixtures, and scheduling, while flame-propeller optimization (MFO) addresses nonlinear challenges in high-rise, energy-efficient structures. The whale optimization algorithm (WOA) addresses multi-objective and constrained problems and, along with other metaheuristic algorithms, facilitates robust global search and sustainable decision-making in civil, material, and structural engineering [90].

## 3. Results and Discussion

This study presents a framework using isolation forest, data normalization, and optimized ensemble models to predict pozzolanic concrete strength, with ET achieving the best performance and multi-objective optimization identifying high-strength, low-CO_2_ mixes. Table 4 summarizes the optimized hyperparameters that enhance prediction accuracy, capture complex patterns, and ensure reliable compressive strength predictions.

Table 4 summarizes the optimized hyperparameters of ensemble ML models for predicting CS of pozzolanic concrete, optimized to enhance robustness, accuracy, and generalization. ET and RF use 100 trees with controlled depths (15–16) and minimum samples per split and leaf (2 and 1) to balance bias and variance, while BD employs 50 estimators with full data and feature sampling to enhance stability. GB and AB employ 100 estimators with a learning rate of 0.1, utilizing deep base trees to capture complex nonlinear interactions while mitigating overfitting. XG uses 100 trees (max_depth = 19) with full data and feature subsampling and regularization (reg_lambda = 1) for robustness, while LG employs 100 estimators with 31 leaves, controlled depth, and bagging fractions for efficient, reliable learning. These optimized hyperparameters allow the models to capture complex patterns in pozzolanic concrete and provide accurate, robust, and generalizable predictions while minimizing bias and overfitting. Parameters such as tree number, depth, and minimum samples control capacity and overfitting, while learning rate, feature sampling, and leaves affect contribution, correlation, and efficiency; regularization enhances robustness and stability. Table 5 shows ensemble ML model performance across normalization techniques.

Table 5 compares seven ensemble ML models across four normalization methods. ET consistently exhibits superior performance, achieving the lowest training errors (MSE = 1.549, RMSE = 1.244, R^2^ = 0.994), minimal errors on the full dataset (MSE = 1.480, RMSE = 1.216, R^2^ = 0.994), and robust testing results (MSE = 17.615–30.217, RMSE = 4.197–5.497, R^2^ = 0.950–0.978), demonstrating exceptional learning capacity, stability, and generalization across all normalization techniques. RF exhibits strong predictive performance across all normalization techniques, with training RMSE of 2.788–2.802 (R^2^ ≈ 0.972–0.973), testing RMSE of 6.400–6.428 (R^2^ = 0.912–0.932), and consistent full-dataset metrics (RMSE ≈ 2.683–2.686, R^2^ = 0.973), indicating robust learning and reliable generalization. BD demonstrates comparable performance, with slightly lower training sensitivity (RMSE 2.772–2.785, R^2^ ≈ 0.972) and testing RMSE of 6.369–6.411 (R^2^ = 0.904–0.925), sustaining consistent results across all datasets. RF exhibits slightly superior testing generalization, while BD demonstrates more consistent stability, underscoring their robustness for predictive applications in structural and civil engineering. XG demonstrates robust performance, with training RMSE of 1.875, testing RMSE of ≈ 7.090, all-dataset RMSE of 2.054, and R^2^ = 0.984–0.987, indicating consistent learning and stable generalization across normalization techniques. GB and LG exhibit moderate predictive capability, with GB showing training RMSE of 2.540, testing RMSE 7.231–7.348, and all-dataset RMSE 2.716, while LG achieves training RMSE 3.312–3.362, testing RMSE 6.061–6.406, and all-dataset RMSE 3.162–3.168, reflecting moderate generalization and sensitivity to unseen data. AB shows the lowest prediction performance, with training RMSE ≈ 8.928–9.011, testing RMSE 8.771–9.092, total dataset RMSE 7.464–7.595, and R^2^ = 0.65–0.71, indicating persistent poor performance and limited generalization. The standard scaler and the min–max scaler generally improve model generalization with lower test RMSEs, while the robust scaler and the max–max scaler slightly increase the errors for XG and GB, indicating a decrease in scaling effectiveness for the boosting models. The standard scaler standardizes features by centering them to a zero mean and scaling to unit variance, thereby enhancing model convergence, mitigating the dominance of features with larger magnitudes, and ensuring consistent performance across algorithms [86]. To rigorously evaluate its reliability, regression performance metrics were computed for multiple ensemble models across training, test, and complete datasets (Table 5). The results indicate consistently high predictive accuracy, with ET achieving the most favorable performance (R^2^ = 0.994, RMSE = 1.216 MPa on the full dataset). Metrics including RMSE, MAE, MedAE, CRMSE, SD, and PCC confirm that the standard scaler effectively reduces scale-induced bias, stabilizes model behavior, and facilitates robust, generalizable predictions. Its efficacy is well-documented in regression and ensemble learning contexts, particularly for datasets with heterogeneous units or ranges, although its performance may be compromised by extreme outliers or markedly non-Gaussian distributions [91].

**Figure 6:**
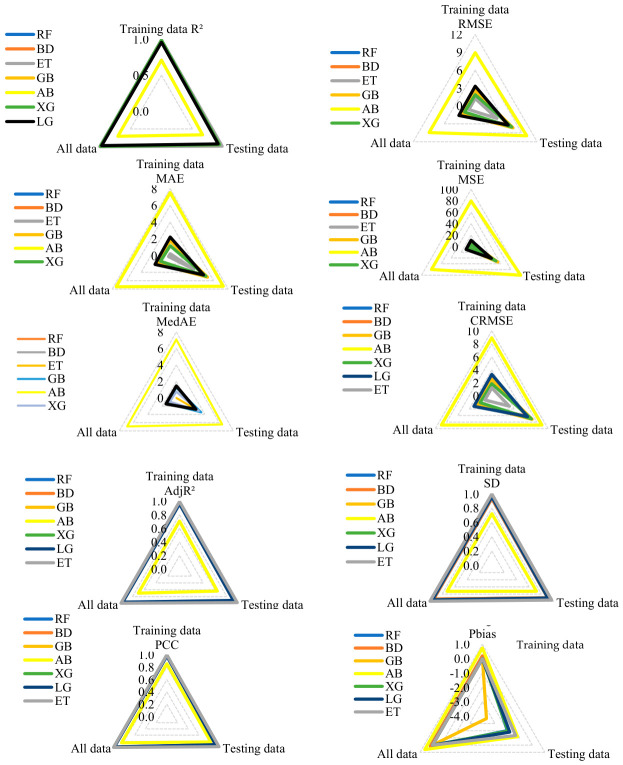
Comparative performance of ensemble ML models using standard scaler.

**Figure 7:**
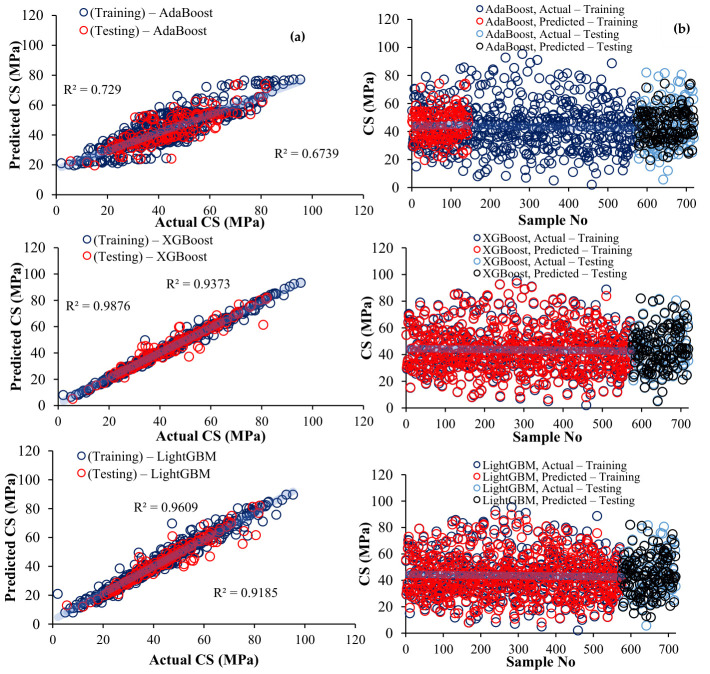

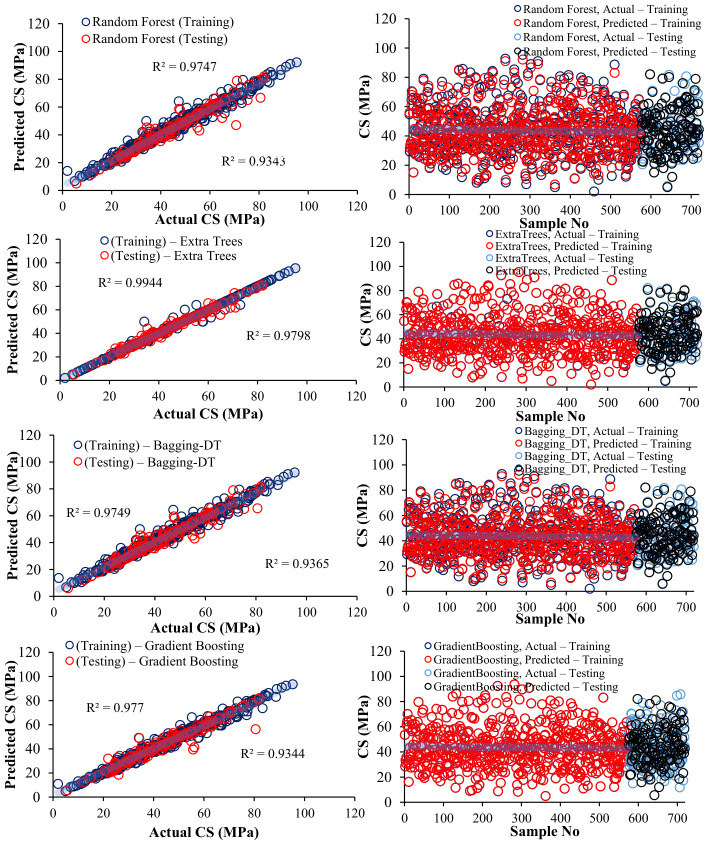
Comparative analysis of CS predictions: (**a**) predicted–actual relationships, (**b**) actual vs. predicted values across samples.

Figure 6 compares ensemble ML model performance with the standard scaler across training, testing, and full datasets. ET demonstrates the highest predictive accuracy with minimal error, followed by BD and RF, which exhibit stable and consistent performance. Boosting models (XG, GB, AB, LG) exhibit variable generalization, with AB underperforming and GB showing pronounced train–test discrepancies. Using the standard scaler enhances model stability through consistent scaling of features, supporting robust performance evaluation, and establishing ET as the benchmark prediction model. Figure 7 presents a comparative analysis of CS predictions, depicting (Figure 7a) predicted–actual relationships and (Figure 7b) actual versus predicted values across the sample set. A sensitivity analysis of outlier removal methods revealed that the ET model achieved a test R^2^ of 0.940 (RMSE = 6.50 MPa, MAE = 4.32 MPa) without any outlier elimination, whereas selective removal using isolation forest (5%) enhanced performance to R^2^ = 0.978 (RMSE = 4.20 MPa, MAE = 3.23 MPa). In comparison, IQR (36.36% removed) and Z-score (8.03% removed) approaches resulted in lower predictive accuracy, with R^2^ = 0.956 (RMSE = 5.98 MPa, MAE = 4.20 MPa) and R^2^ = 0.965 (RMSE = 4.80 MPa, MAE = 3.80 MPa), respectively, demonstrating that moderate, data-driven outlier elimination optimizes model performance while maintaining engineering relevance.

Figure 7 presents a comparative analysis of CS predictions, encompassing predicted–actual relationships and sample-wise prediction patterns. Close alignment along the diameter indicates high prediction accuracy, with ET, XG, and RF showing the strongest agreement with the true values. The deviations indicate prediction errors, with ET recording the most accurate power changes, AB performing the poorest, and BD and LG generally showing good alignment. This figure highlights the stability and robustness of tree-based ensemble models and identifies ET as the most effective predictor.

**Figure 8:**
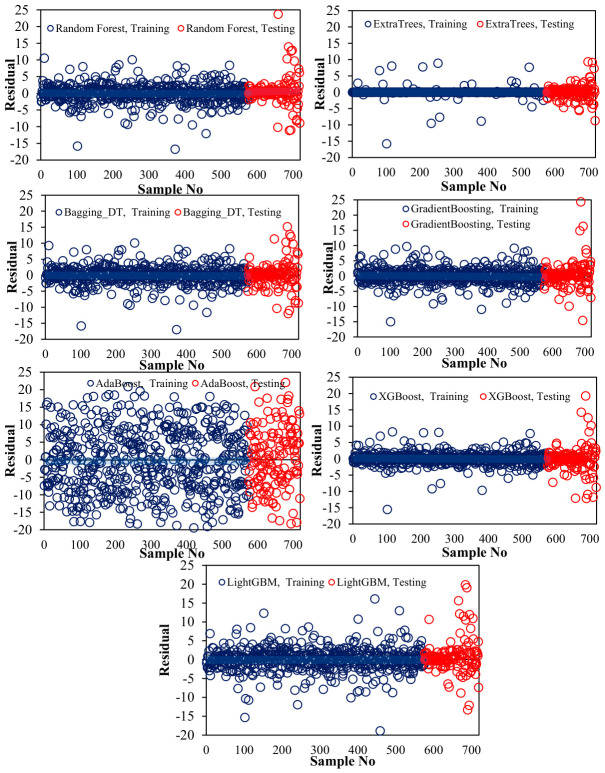
Residual distribution across samples.

Figure 8 shows the residual distribution of CS predictions across samples, where the residuals represent the deviation between the predicted and actual values. ET shows narrow, uniformly distributed residuals and minimal systematic bias, indicating high prediction accuracy, while BD and RF show moderate residuals with occasional fluctuations, and LG and GB show greater dispersion. AB shows the largest residuals, indicating poor prediction performance, while XG maintains the controlled residual patterns. Residual analysis highlights the relative accuracy and error patterns of each model, confirming the robustness of tree-based groups and the superior performance of ET.

**Figure 9:**
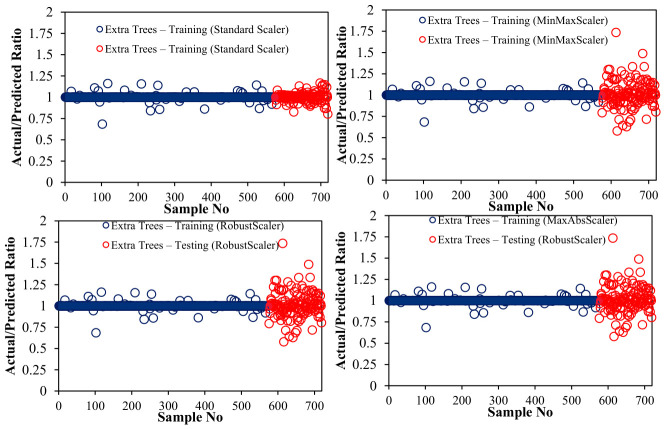
Actual-to-predicted SC ratio of ET with different scalers.

Figure 9 indicates the ratio of actual to predicted CS for ET at different normalization scales and assesses the accuracy of the predictions. Ratios close to 1.0 indicate high prediction accuracy, with the standard closest alignment scaler, the partial dispersion robust scaler, the minimum maximum average variability scaler, and the maximum deviations scaler showing relatively lower consistency. This analysis highlights the impact of normalization on ET predictions and confirms its robust performance, with standard scaling providing the most consistent and accurate results.

**Figure 10:**
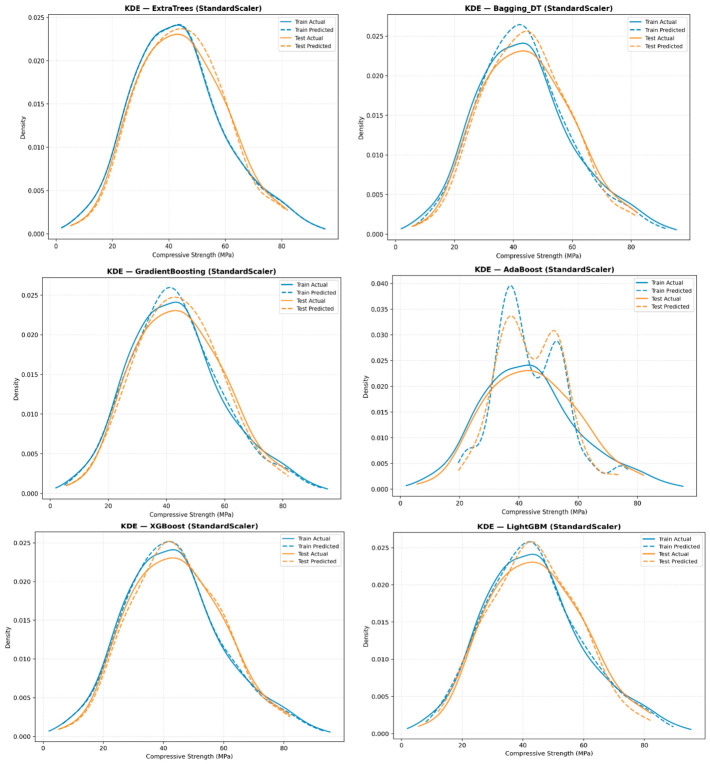

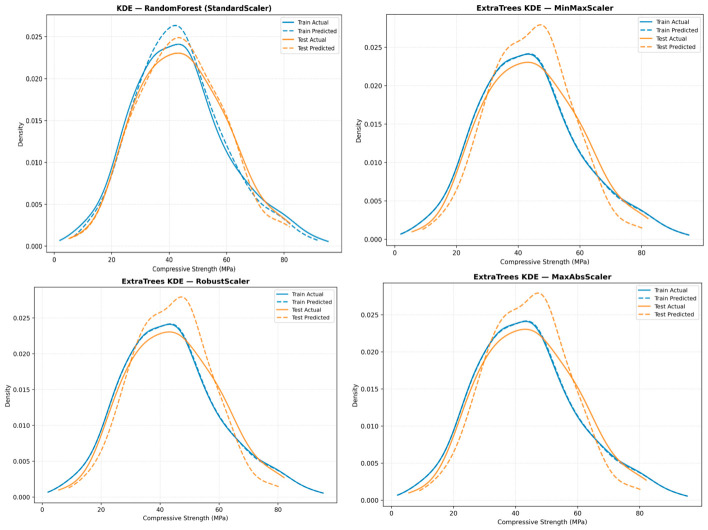
Kernel density estimation of CS predictions obtained from ensemble ML models.

**Figure 11:**
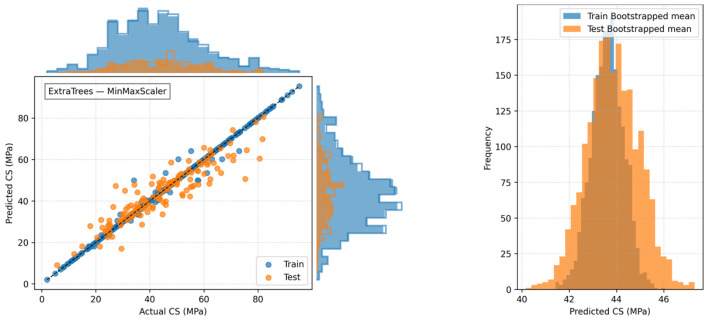

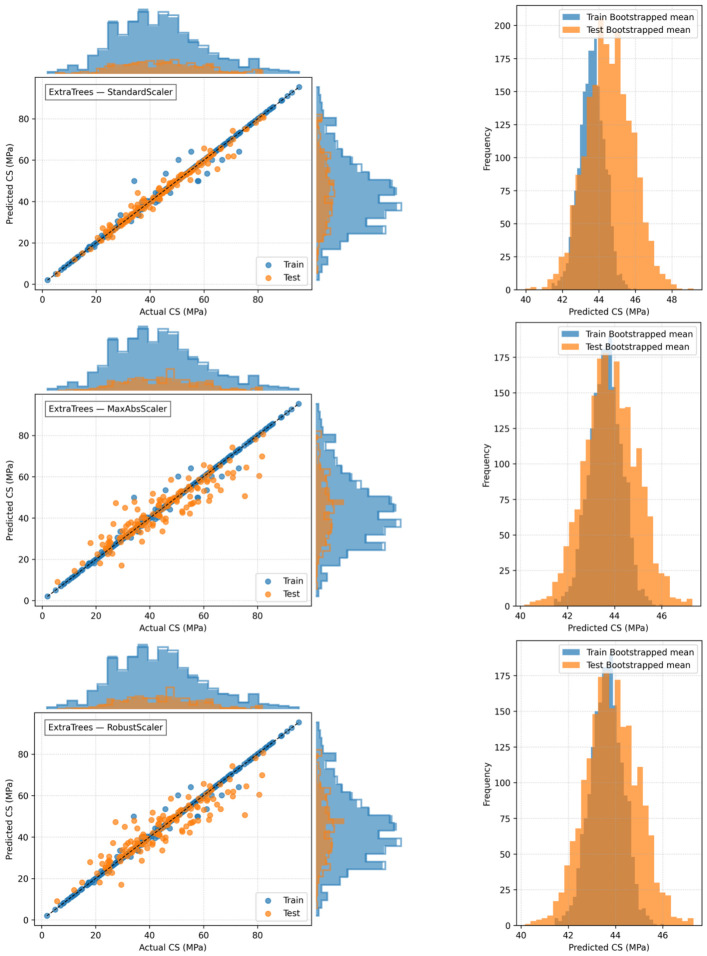
Predicted vs. actual CS (MPa) for ET with marginal distributions and bootstrap intervals.

Figure 10 shows the kernel density estimation (KDE) of CS predictions from the ensemble ML models, which sheds light on the distribution patterns and consistency of predicted concrete strengths. ET shows a narrowly focused density peak aligned with the true power distribution, indicating high prediction accuracy and minimal variance. BD, RF, and XG show a relatively flat density distribution, while GB shows a wider dispersion, indicating increased variability in the predictions. AB shows an irregular and highly scattered density distribution, indicating poor forecast performance, while LG shows stable and smooth densities, indicating consistent forecast behavior. KDE analysis reveals model-specific prediction patterns and confirms the superior predictive performance of ET. Extra trees using the standard scalar exhibit a highly concentrated and well-aligned density peak, indicating a closer match to the true resistivity distribution compared to other normalization techniques. Figure 11 presents the predicted and actual CS values for ET at different scales, including marginal distributions and bootstrap confidence intervals, to assess the prediction accuracy and uncertainty under different normalization techniques.

To ensure robust model evaluation, preprocessing was applied exclusively to the training set to prevent information leakage, and 10-fold cross-validation was used, with metrics averaged across folds to minimize sensitivity to partitioning and hidden clustering. Despite the absence of leave-one-source-out validation, the consistent performance across training, testing, and cross-validation demonstrates strong generalization, and we acknowledge that source-grouped validation could further reinforce these findings.

Figure 11 presents predicted and actual CS for ET across different scalers, incorporating marginal distributions and bootstrap intervals to evaluate prediction accuracy and associated uncertainty. The bivariate plot shows the alignment between predicted and actual values, where the standard scaling shows the closest match and most symmetrical distributions. The robust scaler, the min–max scaler, and the max–abs scaler show slightly wider dispersion and wider bootstrap intervals. Marginal histograms show the agreement between the predicted and actual distributions, while bootstrap intervals capture the variability and uncertainty of the predictions. This figure shows the impact of scaling on ET accuracy, distribution patterns, and predictive power, confirming standard scaling as the most effective normalization approach.

### 3.1. Cross Validation

Cross-validation assesses model performance by partitioning data into k-folds, training on k-1 folds, and validating on the remaining fold iteratively [92]. This approach improves generalizability, provides accurate estimates, and reduces overfitting, especially for limited datasets. In material and civil engineering, cross-validation is used to predict material properties and support structural health monitoring [93]. This facilitates the selection of the optimal model, assesses stability and robustness, and ensures reliable and generalizable predictions for risk-based engineering decisions [94]. Table 6 presents a comparative assessment of BD, ET, RF, GB, AB, XG, and LG under four normalization techniques (standard scaler, max–abs scaler, min–max scaler, robust scaler), with performance evaluated using MAE, MedAE, R^2^, MSE, RMSE, Pbias, CRMSE, SD, and PCC. RF shows strong and stable predictive performance across all normalization techniques and achieves high accuracy and strong correlation (standard scaler: MSE = 45.293, RMSE = 6.626, MAE = 4.611, R^2^ = 0.920, PCC = 0.929), which is comparable to the results obtained under the min–max scaler, robust scaler, and max–abs scaler (RMSE 6.518–6.540, R^2^ 0.904–0.919, PCC > 0.915). The BD model shows slightly higher errors than RF, but maintains its competitive performance, achieving RMSE = 6.852, R^2^ = 0.919, and PCC = 0.923 using the standard scaler, as well as comparable results across all normalization techniques (RMSE 6.826–6.877, R^2^ 0.898–0.917, PCC ≈ 0.910), indicating the variance-reducing effect of group averaging. ET achieves the lowest RMSE and MSE among all models, especially with the standard scaler (RMSE = 5.053, MSE = 25.752) and the min–max scaler (RMSE = 5.713, MSE = 33.784). R^2^ values between 0.926 and 0.966 indicate strong explanatory power, while MedAE values between 2.393 and 2.665 indicate ET’s robustness to outliers and its capacity to minimize median prediction errors. GB shows moderate performance (RMSE ≈ 7.17, R^2^ = 0.910–0.925) with higher errors than ET and RF, while AB has a poorer performance (RMSE > 9.4, R^2^ ≥ 0.647), indicating lower accuracy and greater sensitivity to outliers. XG shows strong performance (RMSE 6.114–6.343, R^2^ 0.906–0.937, PCC > 0.920), indicating stability across scaling, while LG shows moderate but reliable performance (RMSE 6.336–6.355, R^2^ 0.902–0.912, PCC ≈ 0.920), slightly lower than RF, ET, and XG. Across all models, scaling minimally affects ET, RF, and XG, reflecting inherent robustness to feature normalization, whereas AB is more sensitive, indicating potential benefits from hyperparameter tuning or tailored preprocessing. Comparisons of MAE and MedAE show that ET and RF are particularly robust, maintaining low median errors and reliable predictions despite the presence of outliers. CRMSE and SD analyses further confirm that ET, RF, and XG residual distributions exhibit stable distributions with minimal dispersion, while PCC values above 0.91 indicate strong linear correlation and predictive reliability. Table 6 shows that ensemble methods, especially ET, XG, and RF, achieve low errors, high correlation, and consistent performance in scaling techniques, which supports their use in engineering applications that require stability and generalization.

**Figure 12:**
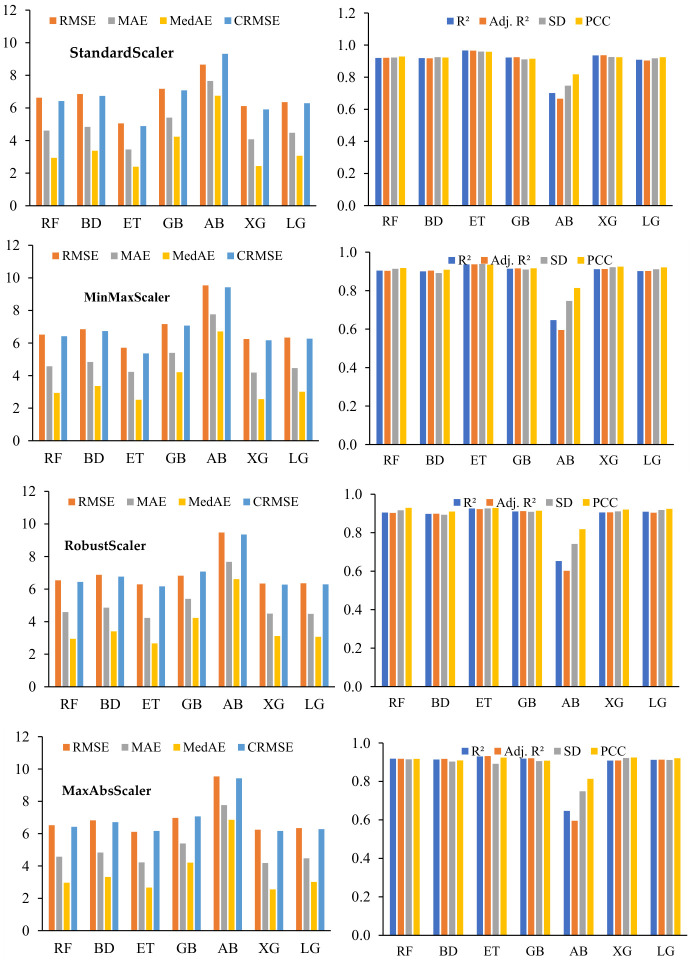
Average 10-fold cross-validation performance of ensemble ML models across scalers.

Figure 12 depicts the average 10-fold cross-validation performance of ensemble ML models across different scalers, highlighting their predictive accuracy and stability. ET consistently achieves the highest average R^2^ and lowest errors across all scalers, demonstrating superior reliability, whereas RF and BD maintain stable, competitive performance, and GB shows moderate results with greater variability. XG and LG exhibit strong performance but moderate sensitivity to scaling, whereas AB shows a pronounced decline in predictive accuracy. Standard scaler optimizes tree-based model performance, robust scaler enhances outlier robustness, and ET remains the most reliable, highlighting the importance of scaler selection.

**Figure 13:**
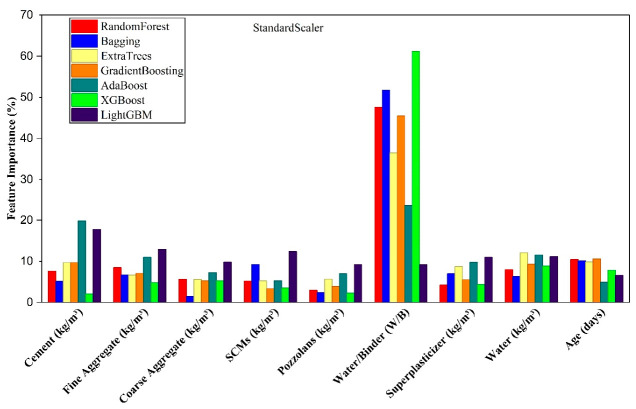
Feature importance of input variables determined by the ensemble ML model using standard scaler.

Figure 13 illustrates the relative importance of the input variables as quantified by the ensemble ML model trained with a standard scaler, highlighting the features that most substantially influence the prediction of CS. Across all models, the W/B exhibits the greatest relative importance, exerting a predominant regulatory influence on CS. In contrast, cement, water, and curing age display moderate yet significant contributions, collectively governing the kinetics of hydration and the progressive, time-dependent development of mechanical strength. SCMs, pozzolans, aggregates, and superplasticizer content exhibit only marginal influence on CS. The model ranks features by their contributions to predictive fidelity, corroborating expected material–property relationships and guiding rational mix optimization.

**Figure 14:**
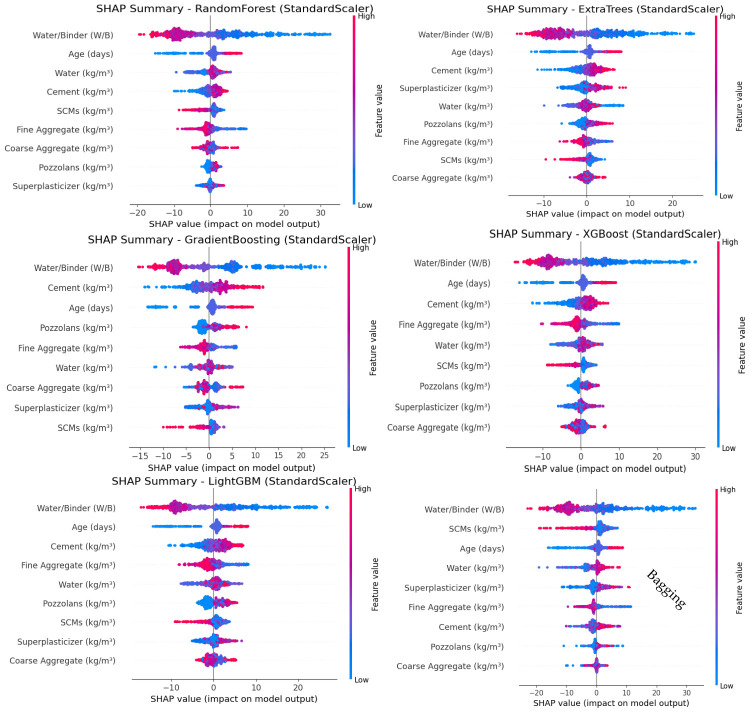
SHAP summary of input feature contributions to ensemble ML-based CS prediction.

Figure 14 illustrates a SHAP summary plot that quantifies the relative contributions of input features to the ensemble ML-based prediction of CS. Each graph quantifies the influence of input features on CS predictions, and SHAP values reflect both the magnitude and direction of their effects. The horizontal axis represents SHAP values, the vertical axis lists the input features, and the magnitude of the features is displayed via a color gradient from blue to red, from low to high values. The W/B illustrates the most significant detrimental effect on the predicted CS, while more cement and longer curing age exert beneficial effects by increasing hydration kinetics and promoting gradual microstructural densification. SCMs and pozzolans show moderate and model-dependent effects on CS, while fine and coarse aggregates have a moderate but consistent effect, and superplasticizer contributes significantly only under specific modeling conditions. Variations in feature rankings and SHAP magnitude indicate different sensitivities in ensemble algorithms, while SHAP analyses enhance interpretability by elucidating nonlinear interactions and confirming the importance of key composition parameters.

**Figure 15:**
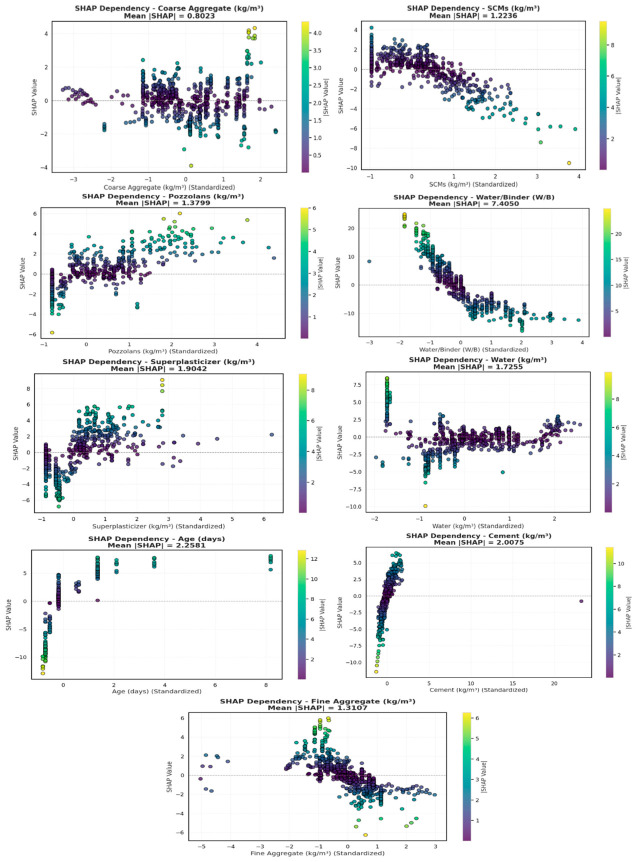
SHAP dependency plot of input features for CS prediction.

**Figure 16:**
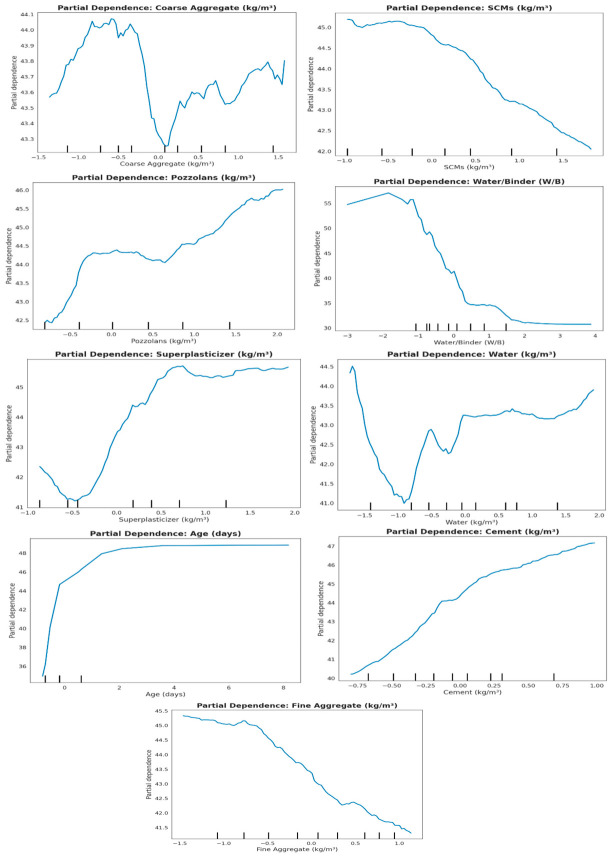
Partial dependence plots of input features for CS prediction.

Figure 15 shows SHAP dependency plots for input features in CS prediction, showing how standard feature values modulate model outputs, with a color gradient from blue to yellow indicating increasing feature magnitude. W/B ratio presents the greatest negative effect on CS, as indicated by its highest mean SHAP value of 7.4050, while cement and curing age have significant positive contributions, with mean SHAP values of 2.0075 and 2.2581, respectively, thus reaffirming their essential role in controlling strength development. SCMs and pozzolans display moderate, directionally distinct effects on predicted CS, with SCMs generally attenuating strength (mean SHAP = 1.2236) and pozzolans imparting a modest enhancement (mean SHAP = 1.3799). Superplasticizer (mean SHAP = 1.9042) and water (mean SHAP = 1.7255) have moderate but variable effects on predicted CS, while fine and coarse aggregates have relatively minor but systematically consistent effects. Dependency plots clarify nonlinear relationships and interactions and facilitate a visual and quantitative integrated assessment of their impact on the predicted CS, where W/B, cement, and age appear as the main determinants, while other mix components have secondary contributions. Figure 16 shows partial dependency plots that illustrate the effects of key properties on CS and support SHAP-based mix optimization.

Figure 16 presents PDPs for the input features, depicting their isolated marginal effects on predicted CS while averaging over all other variables, thereby offering a clear and quantitative assessment of feature contributions. Coarse aggregate (CA) exhibits a moderate, non-linear influence on CS, with a slight rise up to a normalized value of approximately –0.5, followed by a decline and stabilization, indicating that both insufficient and excessive CA contents can marginally impair strength. SCMs impart a predominantly negative influence on predicted CS owing to dilution of the cementitious matrix, whereas pozzolans exert a positive effect by fostering supplementary hydration products through pozzolanic activity. W/B exerts a significant negative effect on CS through increased porosity, while superplasticizer shows a nonlinear beneficial effect, reducing strength at low doses but increasing it at higher levels, thus emphasizing the importance of optimal additive dosage. Water exhibits a complex and non-uniform response reflecting interactions with other mixture components, and intermediate levels optimize predicted strength. Curing age significantly increases CS, and its amount decreases with time, while cement provides an almost linear and stable strength increase, indicating its essential role as the primary binder. Fine aggregate (FA) generally has an adverse effect on CS, as its high proportions exacerbate the demand for paste and reduce the effective binder fraction, thereby reducing overall strength. PDPs exhibit linear and nonlinear effects, in which age, cement, and W/B are the main determinants of CS, while SCMs, pozzolans, aggregates, superplasticizer, and water exert moderate or context-dependent effects, supporting model interpretability and mix optimization.

**Figure 17:**
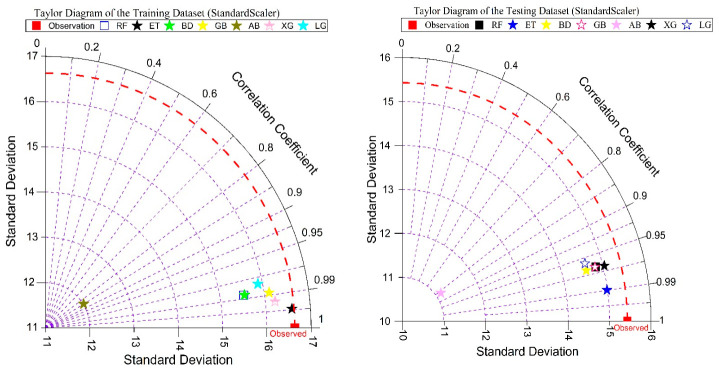
Taylor diagram of ensemble ML model performance for training and testing.

Figure 17 shows a Taylor diagram that evaluates the performance of ensemble ML models on training and test datasets, combining the correlation and standard deviation metrics for a comprehensive evaluation. ET shows the closest match to the reference, indicating the highest prediction accuracy and strongest correlation, while XG and RF show strong but relatively weaker performance. GB and LG achieve competitive performance with marginal deviations, bagging shows moderate matching, while AB is the least efficient, showing the lowest correlation and the highest deviation. Figure 17 underscores predictive concordance, model consistency, and pattern-tracking capability, corroborating the superiority of tree-based ensemble methods and furnishing a robust foundation for model selection and deployment.

The carbon footprint of each optimized mix was assessed via a cradle-to-gate life cycle assessment (LCA). Emission factors for cement, SCMs, aggregates, water, and superplasticizers were sourced from established literature [95]. Total CO_2_ emissions per cubic meter were calculated by summing the products of material quantities and their respective emission coefficients, enabling a transparent and reproducible evaluation of the trade-off between CS and embodied carbon. Table 7 presents the optimal concrete mixing designs obtained through the five meta-heuristic algorithms PSO, DE, GA, MFO, and WOA, along with the predicted CS and associated CO_2_ emissions. Cement varies from 330.715 to 471.695 kg/m^3^, with fine and coarse aggregates adjusted to optimize workability and strength. SCMs and pozzolans are incorporated to improve hydration and long-term performance, while superplasticizer and water are optimized to regulate the W/B ratio. Curing age varies from 30 to 364 days, controlling the time-dependent development of strength. Predicted CS ranges from 88.261 to 91.595 MPa, with DE attaining the highest strength, while CO_2_ emissions vary between 328.46 and 450.68 kg/m^3^, with PSO yielding the lowest footprint. The CO_2_-to-CS ratio illustrates the trade-off between performance and sustainability, demonstrating that metaheuristic optimization effectively balances strength and environmental impact, guiding sustainable high-performance concrete mix design.

In addition to confirming known trends, the analyses reveal nonlinear interactions among key features. The W/B ratio exerts the strongest negative influence on predicted compressive strength, whereas cement content and curing age provide positive contributions. Notably, higher cement content mitigates the adverse effects of elevated W/B ratios, and extended curing age amplifies hydration, partially offsetting porosity induced by high water content. These interactions, observed in SHAP and partial dependence plots, provide mechanistic insights into binder availability, hydration kinetics, and microstructural densification, thereby informing optimized, data-driven mix design.

### 3.2. Validation of Extra Trees Model Performance

The ET model was evaluated using an 80–20% train–test split and multiple statistical metrics (RMSE, R^2^, MAE, MSE, MedAE, Pbias, CRMSE, SD, PCC), ensuring a rigorous assessment of predictive performance. It consistently outperformed other ensemble models and normalization methods across testing and cross-validation. Interpretability analyses (feature importance, SHAP, PDP) identified W/B ratio, curing age, and cement content as dominant factors, consistent with concrete science. Comparison with existing studies (Table 8) further confirms the robustness and reliability of the proposed framework.

Table 8 presents a comparative assessment of the predictive performance of the current ET model relative to a wide range of previously published studies on concrete compressive strength. The present study, based on a dataset of 759 samples and an 80:20 train–test split, demonstrates exceptional predictive performance, attaining a training R^2^ of 0.994 and a testing R^2^ of 0.978, with low prediction errors as reflected by RMSE and MAE values. Existing studies indicate that the predictive performance of models such as ANN, RF, GEP, SVM, and XG is contingent upon factors including dataset size, train–test partitioning, and model architecture. Notably, advanced ML approaches such as RF-PSO and GB-PSO attain high predictive accuracy (R^2^ > 0.98), effectively capturing complex non-linear relationships, whereas traditional regression techniques and simpler ANN architectures exhibit comparatively moderate performance. Advanced metaheuristic models, such as GA+GEP and AB-PSO, can provide optimal forecasting performance; however, they may have higher RMSE under certain conditions, indicating their significant sensitivity to parameter configuration. Ensemble tree-based methods, especially ET, consistently outperform single-model and hybrid approaches in accuracy and robustness, confirming their superiority for predicting concrete compressive strength.

All ensemble models, including ET, were optimized via extensive hyperparameter tuning, testing numerous combinations to achieve the best predictive performance while maintaining generalization. Table 4 summarizes the selected hyperparameters for each model. ET achieved the highest training (R^2^ = 0.994) and testing (R^2^ = 0.978) performance, with a maximum depth of 16. Comprehensive diagnostics, encompassing predicted–actual relationships, residual analysis, kernel density estimation, and bootstrap intervals (Figure 7, Figure 8, Figure 9, Figure 10 and Figure 11), affirm the ET model’s stability and unbiased predictive performance, indicating that its high accuracy reflects genuine generalization rather than overfitting.

While ML optimization approaches exist in prior concrete research, this study emphasizes the rigorous integration of ensemble ML models, comprehensive preprocessing, interpretable analyses, and multi-objective metaheuristic optimization. The framework combines seven ensemble models on nine critical mix parameters, multi-method outlier detection and normalization, SHAP/PDP-based interpretability, and sustainability-driven optimization (DE, GA, MFO, PSO, WOA) to enable robust, transparent, and generalizable prediction and design of high-performance, low-carbon concrete.

## 4. Conclusions

This study proposes a robust ensemble ML framework to predict the CS of pozzolanic concrete using nine key mix parameters. The methodology included rigorous statistical preprocessing, multi-method outlier detection, and evaluation of seven ensemble algorithms under various normalization strategies. Interpretability and validation were ensured through feature importance, PDP, SHAP analyses, Taylor plots, 10-fold cross-validation, and comparison with previous studies, supporting transparent and scientifically rigorous predictions. Limitations include dataset scope, absence of microstructural and chemical descriptors, and lack of experimental validation, which may constrain generalizability. Future work should focus on expanding the dataset, incorporating microstructural and durability parameters, exploring hybrid or physics-informed ML approaches, and experimentally validating optimal mixtures. These steps will enhance industrial applicability and promote sustainability in pozzolanic concrete design.

The principal conclusions derived from this study are summarized as follows:

1. ANOVA confirmed that cement, water, SCMs, pozzolans, W/B, superplasticizer, aggregates, and curing age exert statistically significant effects on CS of concrete, while advanced outlier detection techniques, including isolation forest, and normalization strategies, with standard scaler performing optimally, were applied to ensure data integrity and maximize predictive model performance.
2. Among seven ensemble ML models, extra trees with a standard scaler demonstrated superior predictive accuracy and robustness, attaining training R^2^ = 0.994 (RMSE = 1.244 MPa, MAE = 0.231 MPa), testing R^2^ = 0.978 (RMSE = 4.197 MPa, MAE = 3.234 MPa), and 10-fold cross-validated R^2^ = 0.966 (RMSE = 5.053 MPa, MAE = 3.452 MPa), highlighting its ability to capture the complex nonlinear interactions governing pozzolanic concrete compressive strength.
3. Analyses based on SHAP, feature importance, and partial dependence revealed that W/B ratio, age, and cement constitute the principal determinants of compressive strength, with W/B exerting the strongest negative effect and age and cement the most significant positive contributions, while other constituents exerted moderate or context-dependent influences, elucidating complex mixture interactions and informing optimized mix design.
4. ML-driven sustainability optimization yielded low-carbon, high-strength concrete, with DE achieving 91.60 MPa CS, PSO minimizing carbon intensity (3.65 kg CO_2_/MPa at 90 MPa), and other algorithms providing balanced solutions, highlighting efficient SCM and pozzolan utilization for sustainable mix design.
5. This study integrated advanced outlier detection, feature normalization, and ensemble ML modeling with comprehensive interpretability analyses, residuals, KDE, bootstrap, and Taylor diagrams and sustainability-driven optimization, confirming the stability, unbiased error structure, and superior predictive performance of the ET model, addressing prior limitations in dataset dimensionality, preprocessing, and interpretability, and demonstrating adaptability and scalability for broader application to advanced cementitious materials.

## Figures and Tables

**Table 1:** Assessment of outlier detection techniques and their effects on the concrete dataset.

Method	Outliers R276.	Percentage (%)
IQR	276	36.36%
Z-Score	61	8.03%
Modified Z-Score	313	41.24%
LOF	38	5.01%
DBSCAN	138	18.17%
KNN Distance	38	5.01%
Isolation Forest	38	5%
One-Class SVM	39	5.14%

**Table 2:** Descriptive statistics and ANOVA results for variables influencing CS.

Variable	Mean	StDev	Minimum	Median	Maximum	F-Value	*p*-Value
Cement (kg/m^3^)	301.01	95.03	74.00	296.00	620.00	4.02	0.000
Fine Aggregate (kg/m^3^)	817.82	151.67	30.00	835.60	1221.00	4.67	0.000
Coarse Aggregate (kg/m^3^)	899.10	125.59	458.00	908.00	1226.00	6.78	0.000
SCMs (kg/m^3^)	63.56	66.72	0.00	49.60	330.00	2.10	0.000
Pozzolans (kg/m^3^)	58.64	70.43	0.00	34.00	330.00	1.28	0.016
Water/Binder (W/B)	0.436	0.12	0.20	0.420	0.820	16.88	0.000
Superplasticizer (kg/m^3^)	3.18	3.52	0.00	1.630	18.300	1.52	0.000
Water (kg/m^3^)	175.54	29.26	116.50	174.50	247.00	3.43	0.000
Age (days)	33.67	33.10	3.00	28.00	365.00	10.26	0.000
Compressive Strength (MPa)	43.83	16.39	2.00	43.16	95.330		

**Table 3:** Metrics for regression model evaluation [80].

Abbreviation	Mathematical Formula	Description
$R^{2}$	$1-\frac{\sum_{i=1}^{n}{\left(y_{i}-{\hat{y}}_{i}\right)}^{2}}{\sum_{i=1}^{n}{\left(y_{i}-\bar{y}\right)}^{2}}$	Coefficient of determination; values near 1 indicate better performance.
RMSE	$\sqrt{\frac{1}{n}\sum_{i=1}^{n}{\left(y_{i}-{\hat{y}}_{i}\right)}^{2}}$	Root mean squared error; smaller values indicate higher accuracy.
MAE	$\frac{1}{n}\sum_{i=1}^{n}|y_{i}-{\hat{y}}_{i}|$	Mean absolute error; smaller values indicate more accurate predictions.
$\mathrm{Adj}\;R^{2}$	$1-\frac{\left(1-R^{2})(n-1\right)}{n-p-1}$	Adjusted R^2^ penalizes irrelevant predictors, providing a more accurate measure of explained variance.
PCC	$\frac{\mathrm{Cov}(y,\hat{y})}{\sigma_{y}\sigma_{\hat{y}}}$	Pearson correlation coefficient; values near 1 indicate a strong linear relationship.
SD	$\sqrt{\frac{1}{n}\sum_{i=1}^{n}{\left(y_{i}-\bar{y}\right)}^{2}}$	Standard deviation; indicates data spread, applied in Taylor diagrams.
cRMSE	$\sqrt{\frac{1}{n}\sum_{i=1}^{n}{\left[\left(y_{i}-\bar{y})-({\hat{y}}_{i}-\bar{\hat{y}}\right)\right]}^{2}}$	Centered root mean square difference; smaller values indicate better pattern agreement.
MedAE	$median\;(\left|y_{i}-{\hat{y}}_{i}\right|)$	MedAE (median absolute error); provides a robust measure of model error, less sensitive to outliers.

Note: y_i_ is the observed (actual) value, ${\hat{y}}_{i}$ is the predicted value, $\bar{y}$ is the mean of observed values, $\bar{\hat{y}}$ is the mean of predicted values, n is the number of observations, p is number of predictors, Cov (y, $\hat{y}$) is the covariance between observed and predicted values, and σ_y_ and $\sigma_{\hat{y}}$ are the standard deviations of observed and predicted values, respectively.

**Table 4:** Optimized hyperparameters of ensemble ML Models.

Model	Hyperparameters
Random Forest	n_estimators = 100, max_depth = 15, min_samples_split = 2, min_samples_leaf = 1, max_features = ‘auto’
Bagging_DT	n_estimators = 50, base estimator = Decision Tree Regressor (max_depth = 14), max samples = 1.0, max_features = 1.0,
Extra Trees	n_estimators = 100, max_depth = 16, min_samples_split = 2, min_samples_leaf = 1, max_features = 2
Gradient Boosting	n_estimators = 100, learning_rate = 0.1, max_depth = 18, min_samples_split = 2, min_samples_leaf = 1, subsample = 1.0
AdaBoost	n_estimators = 100, learning_rate = 0.1, base estimator = Decision Tree Regressor(max_depth = 14),
XGBoost	n_estimators = 100, learning_rate = 0.1, max_depth = 19, subsample = 1.0, colsample_bytree = 1.0, reg_lambda = 1
LightGBM	n_estimators = 100, learning_rate = 0.1, num_leaves = 31, max_depth = 15, min_data_in_leaf = 20, feature_fraction = 1.0, bagging fraction = 1.0

**Table 5:** Performance of ensemble ML models under various normalization techniques.

MSE	RMSE	MAE	MedAE	R^2^	AdjR^2^	Pbias	CRMSE	SD	PCC	Model	Scaler	Dataset
7.775	2.788	1.890	1.272	0.972	0.971	0.172	2.787	0.934	0.987	RF	StandardScaler	Train
41.321	6.428	4.616	3.037	0.935	0.934	−2.009	6.365	0.901	0.912	RF		Test
7.187	2.681	1.772	1.098	0.973	0.973	0.164	2.680	0.936	0.988	RF		All data
7.682	2.772	1.872	1.214	0.972	0.972	0.201	2.770	0.935	0.987	BD		Train
41.098	6.411	4.598	3.165	0.925	0. 923	−1.899	6.354	0.90	0.912	BD		Test
7.200	2.683	1.774	1.133	0.973	0.973	0.162	2.682	0.936	0.988	BD		All data
1.549	1.244	0.231	0.000	0.994	0.994	0.000	1.244	0.997	0.997	ET		Train
17.615	4.197	3.234	2.05	0.978	0. 978	−1.34	3.148	0.965	0.974	ET		Test
1.480	1.216	0.240	0.000	0.994	0.994	0.000	1.216	0.997	0.997	ET		All data
6.452	2.540	1.768	1.257	0.977	0.976	0.000	2.540	0.969	0.988	GB		Train
53.996	7.348	5.148	3.500	0.932	0.930	−3.679	7.162	0.912	0.923	GB		Test
7.377	2.716	1.896	1.332	0.972	0.972	0.000	2.716	0.963	0.986	GB		All data
79.707	8.928	7.520	7.084	0.711	0.707	0.737	8.922	0.722	0.854	AB		Train
79.704	8.928	7.324	6.424	0.663	0.640	−1.181	8.912	0.721	0.821	AB		Test
79.729	8.929	7.441	6.901	0.703	0.699	0.612	8.925	0.722	0.848	AB		All data
3.515	1.875	1.172	0.753	0.987	0.987	0.001	1.875	0.975	0.994	XG		Train
50.271	7.090	4.922	3.154	0.937	0.935	−1.952	7.037	0.926	0.934	XG		Test
4.217	2.054	1.344	0.904	0.984	0.984	0.003	2.054	0.970	0.992	XG		All data
10.971	3.312	2.213	1.456	0.960	0.960	0.000	3.312	0.956	0.980	LG		Train
41.043	6.406	4.607	2.757	0.915	0.912	−1.810	6.355	0.907	0.911	LG		Test
10.036	3.168	2.097	1.455	0.963	0.962	0.000	3.168	0.957	0.981	LG		All data
7.807	2.794	1.893	1.272	0.972	0.971	0.151	2.793	0.934	0.987	RF	MinMaxScaler	Train
41.163	6.416	4.609	3.053	0.914	0.912	−2.012	6.353	0.914	0.912	RF		Test
7.202	2.684	1.774	1.119	0.973	0.973	0.160	2.683	0.936	0.988	RF		All data
7.707	2.776	1.872	1.193	0.972	0.972	0.184	2.775	0.935	0.987	BD		Train
40.765	6.385	4.578	3.163	0.925	0.921	−1.911	6.328	0.904	0.913	BD		Test
7.216	2.686	1.777	1.123	0.973	0.973	0.154	2.685	0.936	0.988	BD		All data
1.549	1.244	0.231	0.000	0.994	0.994	0.000	1.244	0.997	0.997	ET		Train
27.001	5.197	4.134	2.545	0.965	0.964	−1.454	5.148	0.955	0.969	ET		Test
1.480	1.216	0.240	0.000	0.994	0.994	0.000	1.216	0.997	0.997	ET		All data
6.452	2.540	1.768	1.257	0.977	0.976	0.000	2.540	0.969	0.988	GB		Train
52.282	7.231	5.048	3.432	0.923	0.924	−3.763	7.033	0.913	0.932	GB		Test
7.377	2.716	1.896	1.332	0.972	0.972	0.000	2.716	0.963	0.986	GB		All data
79.734	8.929	7.513	7.245	0.711	0.707	0.526	8.926	0.731	0.852	AB		Train
76.933	8.771	7.207	6.056	0.674	0.653	−1.182	8.755	0.721	0.829	AB		Test
79.968	8.943	7.495	6.758	0.702	0.698	0.633	8.938	0.728	0.846	AB		All data
3.515	1.875	1.172	0.753	0.987	0.987	0.001	1.875	0.975	0.994	XG		Train
50.271	7.090	4.922	3.154	0.945	0.953	−1.952	7.037	0.935	0.965	XG		Test
4.217	2.054	1.344	0.904	0.984	0.984	0.003	2.054	0.970	0.992	XG		All data
11.305	3.362	2.239	1.483	0.959	0.958	0.000	3.362	0.957	0.980	LG		Train
36.739	6.061	4.412	2.678	0.900	0.910	−1.450	6.027	0.920	0.920	LG		Test
10.020	3.165	2.079	1.405	0.963	0.962	0.000	3.165	0.958	0.981	LG		All data
7.819	2.796	1.897	1.293	0.972	0.971	0.170	2.795	0.934	0.987	RF	RobustScaler	Train
41.205	6.419	4.604	3.003	0.925	0.923	−2.038	6.354	0.923	0.932	RF		Test
7.212	2.686	1.773	1.098	0.973	0.973	0.158	2.685	0.936	0.988	RF		All data
7.721	2.779	1.877	1.219	0.972	0.972	0.200	2.777	0.935	0.987	BD		Train
40.893	6.395	4.578	3.160	0.912	0.912	−1.941	6.336	0.902	0.913	BD		Test
7.214	2.686	1.773	1.133	0.973	0.973	0.155	2.685	0.936	0.988	BD		All data
1.549	1.244	0.231	0.000	0.994	0.994	0.000	1.244	0.997	0.997	ET		Train
28.058	5.297	4.234	2.645	0.952	0.950	−1.654	5.548	0.945	0.959	ET		Test
1.480	1.216	0.240	0.000	0.994	0.994	0.000	1.216	0.997	0.997	ET		All data
6.452	2.540	1.768	1.257	0.977	0.976	0.000	2.540	0.969	0.988	GB		Train
53.673	7.326	5.107	3.471	0.924	0.923	−3.809	7.126	0.900	0.910	GB		Test
7.377	2.716	1.896	1.332	0.972	0.972	0.000	2.716	0.963	0.986	GB		All data
79.943	8.941	7.539	7.224	0.710	0.706	0.977	8.931	0.727	0.852	AB		Train
81.327	9.018	7.392	6.378	0.656	0.633	−0.901	9.009	0.719	0.816	AB		Test
79.386	8.910	7.435	6.930	0.704	0.700	0.395	8.908	0.721	0.849	AB		All data
3.515	1.875	1.172	0.753	0.987	0.987	0.001	1.875	0.975	0.994	XG		Train
50.271	7.090	4.922	3.154	0.932	0.932	−1.952	7.037	0.923	0.935	XG		Test
4.217	2.054	1.344	0.904	0.984	0.984	0.003	2.054	0.970	0.992	XG		All data
10.677	3.268	2.207	1.489	0.961	0.961	0.000	3.268	0.958	0.981	LG		Train
39.171	6.259	4.532	2.597	0.904	0.912	−1.756	6.209	0.917	0.915	LG		Test
10.001	3.162	2.097	1.422	0.963	0.962	0.000	3.162	0.957	0.981	LG		All data
7.852	2.802	1.899	1.272	0.972	0.971	0.138	2.801	0.934	0.987	RF	MaxAbsScaler	Train
40.963	6.400	4.573	3.023	0.932	0.930	−2.128	6.329	0.923	0.934	RF		Test
7.212	2.685	1.773	1.098	0.973	0.973	0.141	2.685	0.936	0.988	RF		All data
7.759	2.785	1.881	1.219	0.972	0.971	0.161	2.785	0.934	0.987	BD		Train
40.569	6.369	4.546	3.120	0.920	0.924	−2.020	6.305	0.900	0.914	BD		Test
7.220	2.687	1.776	1.106	0.973	0.973	0.138	2.686	0.936	0.988	BD		All data
1.549	1.244	0.231	0.000	0.994	0.994	0.000	1.244	0.997	0.997	ET		Train
30.217	5.497	4.234	2.145	0.954	0.955	−1.754	5.148	0.945	0.959	ET		Test
1.480	1.216	0.240	0.000	0.994	0.994	0.000	1.216	0.997	0.997	ET		All data
6.452	2.540	1.768	1.257	0.977	0.976	0.000	2.540	0.969	0.988	GB		Train
53.572	7.319	5.093	3.432	0.913	0.912	−3.793	7.121	0.902	0.923	GB		Test
7.377	2.716	1.896	1.332	0.972	0.972	0.000	2.716	0.963	0.986	GB		All data
81.205	9.011	7.595	7.281	0.706	0.701	0.809	9.004	0.730	0.849	AB		Train
82.670	9.092	7.425	6.126	0.650	0.627	−1.064	9.080	0.703	0.815	AB		Test
79.723	8.929	7.464	6.883	0.703	0.699	0.651	8.924	0.722	0.848	AB		All data
3.515	1.875	1.172	0.753	0.987	0.987	0.001	1.875	0.975	0.994	XG		Train
50.271	7.090	4.922	3.154	0.935	0.936	−1.952	7.037	0.923	0.943	XG		Test
4.217	2.054	1.344	0.904	0.984	0.984	0.003	2.054	0.970	0.992	XG		All data
11.305	3.362	2.239	1.483	0.959	0.958	0.000	3.362	0.957	0.980	LG		Train
36.739	6.061	4.412	2.678	0.890	0.834	−1.450	6.027	0.920	0.920	LG		Test
9.872	3.142	2.064	1.417	0.963	0.963	0.000	3.142	0.958	0.982	LG		All data

**Table 6:** Average performance of ensemble ML models based on 10-fold cross-validation.

MSE	RMSE	MAE	MedAE	R^2^	AdjR^2^	Pbias	CRMSE	SD	PCC	Model	Scaler	Fold
45.293	6.626	4.611	2.941	0.920	0.921	0.886	6.424	0.923	0.929	RF	StandardScaler	Average 10-Fold CV Statistics for ML Models Using Different Scalers
47.955	6.852	4.839	3.374	0.919	0.918	0.901	6.738	0.925	0.923	BD		
25.752	5.053	3.452	2.393	0.966	0.965	0.649	4.891	0.960	0.959	ET		
52.659	7.174	5.408	4.236	0.923	0.925	0.478	7.082	0.911	0.915	GB		
89.838	9.423	7.657	6.747	0.701	0.666	1.109	9.322	0.747	0.818	AB		
38.130	6.114	4.078	2.437	0.936	0.937	0.636	5.909	0.926	0.925	XG		
41.730	6.355	4.477	3.070	0.909	0.904	0.737	6.291	0.918	0.924	LG		
43.819	6.518	4.569	2.937	0.904	0.903	0.844	6.416	0.914	0.918	RF	MinMaxScaler	
47.890	6.847	4.836	3.362	0.900	0.904	0.847	6.735	0.892	0.909	BD		
33.784	5.713	4.227	2.517	0.936	0.937	0.649	5.360	0.939	0.935	ET		
52.543	7.166	5.397	4.206	0.914	0.916	0.461	7.072	0.910	0.916	GB		
92.074	9.542	7.758	6.713	0.647	0.595	1.158	9.426	0.747	0.814	AB		
39.899	6.246	4.185	2.555	0.912	0.913	0.736	6.170	0.922	0.925	XG		
41.326	6.336	4.461	3.007	0.902	0.902	0.677	6.268	0.911	0.921	LG		
44.110	6.540	4.590	2.950	0.905	0.903	0.887	6.439	0.917	0.929	RF	RobustScaler	
48.319	6.877	4.858	3.408	0.898	0.899	0.923	6.767	0.893	0.910	BD		
40.991	6.293	4.227	2.665	0.926	0.923	0.886	6.168	0.926	0.929	ET		
47.258	6.820	5.404	4.231	0.910	0.913	0.480	7.078	0.909	0.914	GB		
91.047	9.473	7.673	6.612	0.653	0.602	1.359	9.353	0.742	0.818	AB		
41.661	6.343	4.495	3.118	0.905	0.906	0.854	6.272	0.911	0.920	XG		
41.730	6.355	4.477	3.070	0.909	0.904	0.737	6.291	0.918	0.924	LG		
43.898	6.525	4.577	2.969	0.919	0.918	0.807	6.422	0.915	0.918	RF	MaxAbsScaler	
47.592	6.826	4.831	3.321	0.914	0.917	0.841	6.711	0.903	0.910	BD		
38.131	6.112	4.227	2.665	0.930	0.932	0.886	6.168	0.892	0.924	ET		
49.386	6.978	5.395	4.206	0.919	0.921	0.457	7.071	0.906	0.908	GB		
92.362	9.540	7.765	6.859	0.647	0.596	1.111	9.425	0.749	0.813	AB		
39.899	6.246	4.185	2.555	0.909	0.909	0.736	6.170	0.922	0.925	XG		
41.492	6.346	4.475	3.018	0.912	0.913	0.675	6.278	0.912	0.921	LG		

**Table 7:** Optimized mix designs, predicted CS, and CO_2_ footprint.

Parameter	DE	PSO	GA	MFO	WOA
Cement (kg/m^3^)	471.695	330.715	400	420	390
Fine Aggregate (kg/m^3^)	417.897	489.744	450	470	430
Coarse Aggregate (kg/m^3^)	927.043	1087.681	1000	980	1020
SCMs (kg/m^3^)	54.884	50	55	60	52
Pozzolan (kg/m^3^)	42.161	48.619	40	35	38
Superplasticizer (SP) (kg/m^3^)	8.098	10	9	7	8.5
Water (kg/m^3^)	176.222	160.032	170	180	165
Age (days)	30	364	90	120	100
Predicted Max CS (MPa)	91.595	90	89	90.987	88.261
Total CO_2_ Footprint (kg CO_2_/m^3^)	450.68	328.46	388.2	402.6	378.05
Total CO_2_ Footprint/CSC	4.92	3.65	4.36	4.43	4.28

**Table 8:** Validation of model predictions against established literature.

Reference	Dataset Size	Train/Test Split	Model	Performance Metrics
Current study	759	80:20	Extra Trees	Training R^2^ = 0.994, RMSE = 1.244, MAE = 0.231 Testing R^2^ = 0.978, RMSE = 4.197, MAE = 3.234 All data R^2^ = 0.994, RMSE = 1.216, MAE = 0.240
Matiur Rahman Raju et al. [33]	482	70:30	RF	Training R^2^ = 0.976, RMSE = 2.84, MAE = 2.05 Testing R^2^ = 0.964, RMSE = 7.81, MAE = 5.89
Ahmadi [96,97]	272	80:20	ANN	R^2^ = 0.97, MAPE = 5.8%
			Regression	R^2^ = 0.926, MAPE = 13.2%
Le [98]	880	83:17	ANN	R^2^ = 0.9956, RMSE = 154.66, MAPE = 7.54%
Tran [99]	258	85:15	Regression	R^2^ = 0.95, MAE = 323.52, RMSE = 565
Guneyisi [100]	314	75:25	GEP	MAPE = 7.49%, RMSE = 228
Ipek [101]	103	75:25	GEP	R^2^ = 0.987, MAPE = 6.43%, RMSE = 85.7
Javed [102]	227	78:22	GEP	R^2^ = 0.98, MAE = 138.7, RMSE = 258
Naser [103]	1245/979	70:30	GA+	MAE = 202, RMSE = 295
			GEP	MAE = 238, RMSE = 340
Ren [104]	180	85:15	SVM	Train: R^2^ = 0.932, MAPE = 14.3%, MAE = 239, RMSE = 314 Test: R^2^ = 0.914, MAPE = 14.5%, MAE = 227, RMSE = 304
Memarzadeh [105]	646/347	85:15	GEP	R^2^ = 0.98, MAE = 242, RMSE = 384
			GEP	R^2^ = 0.98, MAE = 324, RMSE = 464
			ANN	R^2^ = 0.99, MAE = 134, RMSE = 205
			ANN	R^2^ = 0.99, MAE = 163, RMSE = 254
Megahed et al. [106]	674/396/246	80:20	SR	MAPE = 5.856%, RMSE = 552 MAPE = 5.856%, RMSE = 368.2 MAPE = 5.756%, RMSE = 194.9
Kashem et al. [49]			AB-PSO	Training R^2^ = 0.9064, RMSE = 12.113 Testing R^2^ = 0.9062, RMSE = 13.017
			RF-PSO	R^2^ = 0.9879, RMSE = 4.3495 R^2^ = 0.9749, RMSE = 6.7348
			GB-PSO	R^2^ = 0.9913, RMSE = 3.6947 R^2^ = 0.9804, RMSE = 5.9486
Shen et al. [107]			XGBoost	Training R^2^ = 0.90, RMSE = 7.6
			AdaBoost	Training R^2^ = 0.82, RMSE = 13.6
			Bagging	Training R^2^ = 0.78, RMSE = 14.6
Abellán-García, J. [108]			ANN	Training R^2^ = 0.8570, RMSE = 10.534 Testing R^2^ = 0.8100, RMSE = 9.925
Khan et al. [109]			GB	Training R^2^ = 0.8100, RMSE = 9.790
			DT	Training R^2^ = 0.7900, RMSE = 10.33
			RF	Training R^2^ = 0.9100, RMSE = 6.76
Alabduljabbar et al. [110]			GEP	Training RMSE = 6.507 Testing RMSE = 4.762
Nguyen et al. [111]			XGBoost	Training R^2^ = 0.8900, RMSE = 7.86

## Data Availability

The data supporting the findings of this study are available from the corresponding author upon reasonable request.
